# The Morphospace of Consciousness: Three Kinds of Complexity for Minds and Machines

**DOI:** 10.3390/neurosci4020009

**Published:** 2023-03-27

**Authors:** Xerxes D. Arsiwalla, Ricard Solé, Clément Moulin-Frier, Ivan Herreros, Martí Sánchez-Fibla, Paul Verschure

**Affiliations:** 1 Departament of Information and Communication Technologies, Universitat Pompeu Fabra (UPF), 08018 Barcelona, Spain; 2 Complex Systems Lab, Universitat Pompeu Fabra, 08003 Barcelona, Spain; 3 Institut de Biologia Evolutiva (CSIC-UPF), 08003 Barcelona, Spain; 4 Santa Fe Institute, Santa Fe, NM 87501, USA; 5 Institució Catalana de Recerca i Estudis Avançats (ICREA), 08010 Barcelona, Spain; 6 Flowers Research Group, Inria Bordeaux, 33405 Talence, France; 7 Donders Institute for Brain, Cognition and Behavior, Radboud University, 6525 GD Nijmegen, The Netherlands

**Keywords:** consciousness, brain networks, artificial intelligence, synthetic biology, cognitive robotics, complex systems

## Abstract

In this perspective article, we show that a morphospace, based on information-theoretic measures, can be a useful construct for comparing biological agents with artificial intelligence (AI) systems. The axes of this space label three kinds of complexity: (i) autonomic, (ii) computational and (iii) social complexity. On this space, we map biological agents such as bacteria, bees, C. elegans, primates and humans; as well as AI technologies such as deep neural networks, multi-agent bots, social robots, Siri and Watson. A complexity-based conceptualization provides a useful framework for identifying defining features and classes of conscious and intelligent systems. Starting with cognitive and clinical metrics of consciousness that assess awareness and wakefulness, we ask how AI and synthetically engineered life-forms would measure on homologous metrics. We argue that awareness and wakefulness stem from computational and autonomic complexity. Furthermore, tapping insights from cognitive robotics, we examine the functional role of consciousness in the context of evolutionary games. This points to a third kind of complexity for describing consciousness, namely, social complexity. Based on these metrics, our morphospace suggests the possibility of additional types of consciousness other than biological; namely, synthetic, group-based and simulated. This space provides a common conceptual framework for comparing traits and highlighting design principles of minds and machines.

## 1. Introduction

How do we build a taxonomy of consciousness based on evidence from clinical neuroscience, synthetic biology, artificial intelligence (AI) and cognitive robotics? Here, we examine current biologically motivated metrics of consciousness. In view of these metrics, we show how contemporary AI and synthetic systems measure on homologous scales. In what follows, we refer to a phenomenological description of consciousness. In other words, that which can be described in epistemically objective terms, even though aspects of the problem of consciousness may require an ontologically subjective description. Drawing from what is known about the phenomenology of consciousness in biological systems, we build a homologous argument for artificial, collective and simulated systems. For example, in clinical diagnosis of disorders of consciousness, two widely used scales are patient awareness and wakefulness (also referred to as arousal), both of which can be assessed using neurophysiological recordings [1,2]. We extrapolate these metrics to construct a morphospace of consciousness.

The origin of the concept of a morphospace comes from comparative anatomy and paleobiology, where quantitative measures or principal components from clustering methods allow classification in a metric-like space. However, it can also involve a qualitative relation-based approach, as the one we will follow here. A related concept of the so-called *theoretical morphospace*, has also been defined in formal terms, as an *N*-dimensional geometric hyperspace produced by systematically varying the parameter values associated to a given (usually geometric) set of traits [3]. More recently, morphospaces have been used in the study of complex systems, linguistics and biology [4,5,6]. Hence, a morphospace serves as a useful tool to gain insights on design principles and evolutionary constraints, when looking across a large class of systems (or species) that display complex variations in traits. For the problem of consciousness, we construct this morphospace based on three kinds of complexity. These considerations suggest an embodiment-based taxonomy of consciousness [7].

For practical reasons, many experimental paradigms testing consciousness are designed for humans or higher-order primates (see [8,9,10] for an overview of the field). In this article, we argue that metrics commonly associated to biological consciousness can be appropriately extrapolated for conceptualizing behaviors of synthetic and artificial intelligence systems. This is insightful not only for understanding parallels between biological and potential synthetic consciousness, but more importantly for unearthing design principles necessary for building biomimetic technology that could potentially acquire consciousness. As evidenced by several historical precedents, bio-inspired design thinking has been at the core of some of the greatest scientific breakthroughs. For instance, early attempts at aviation in the 19th century were inspired by studying flight mechanics in birds and insects (the term aviation itself is derived from the Latin “avis” for “bird”). In fact, biological flight mechanisms are so sophisticated that their biomimetic implementations are still being actively studied within the field of soft robotics [11]. However, it so happened that rather than coming around to mimicking nature exactly, humanity learnt the basic laws of aerodynamics based on observations from nature and looked for other embodiments of those principles. This in fact, led to the invention of the modern aircraft by the Wright brothers in 1903, leading to a completely new way to build machines that fly than those that exactly mimic nature.

Metrics of consciousness are also the right tools to quantitatively study how human and animal intelligence differs from state-of-the-art machine intelligence. Once again, it is instructive to take a historical perspective on human intelligence as laid out by one of the founders of AI, Allen Newell. In 1994, in his seminal work, “Unified Theories of Cognition” [12]. Newell proposed the following thirteen criteria necessary for building human-level cognitive architectures:

- Behave flexibly as a function of the environment
- Exhibit adaptive (rational, goal-oriented) behavior
- Operate in real-time
- Operate in a rich, complex, detailed environment (that is, perceive an immense amount of changing detail, use vast amounts of knowledge, and control a motor system of many degrees of freedom)
- Use symbols and abstractions
- Use language, both natural and artificial
- Learn from the environment and from experience
- Acquire capabilities through development
- Operate autonomously, but within a social community
- Be self-aware and have a sense of self
- Be realizable as a neural system
- Be constructible by an embryological growth process
- Arise through evolution

Current AI architectures still do not meet all these criteria. On the other hand, although Newell did not discuss consciousness back then, the above criteria are equally relevant to the problem of consciousness. Given current advances in our understanding of neural and cognitive mechanisms of consciousness [9], one may well argue that the problem of consciousness supersedes and even subsumes the problem of biological intelligence. While Newell’s criteria list signatures that are the consequence of human intelligence, for consciousness it is more useful to have a list of functional criteria that underlie the process of consciousness. Below, we will discuss such functional criteria.

Why are these considerations relevant for understanding the direction of today’s AI and the development of new technologies? The field of AI, and particularly neural networks, began as a modest attempt to understand cognition and brain function. It dates back to the 1930s with the first model of neural networks by Nicolas Rashevsky [13], followed by the seminal work of Walter Pitts and Warren McCulloch in 1943 [14] and Frank Rosenblatt’s perceptron in 1958 [15]. Eventually, with the use of analytic methods from statistical physics, those simple models paved the way to understanding associative memory and other emergent cognitive phenomena [16]. Even though artificial neural networks did not quite solve the problem of how the brain works, they led to the discovery of brain-inspired computing technologies such as deep learning systems and powerful technologies for computational intelligence such as IBM’s Watson. These machines process massive volumes of data and are built for intensive computational tasks that the brain may not even be designed for. Yet, in spite of these computational successes, contemporary AI is still challenged in many tasks that human and animal brains seem to perform effortlessly. For that reason, the next frontier in AI and machine intelligence will be closely tied to our advances in understanding the governing principles of consciousness and its various embodiments. This potentially has a bearing on the development of next-generation biomimetic and sentient technologies. Recent work in this direction can be found in [7,17,18,19].

## 2. Biological Consciousness: Insights from Clinical Neuroscience

We begin by reviewing clinical scales used for assessing consciousness in patients with disorders of consciousness. In subsequent sections, we will generalize the complexity measures pertinent to these biological scales and discuss how synthetic systems can be measured on these.

### 2.1. Clinical Consciousness and its Disorders

**Figure 1:**
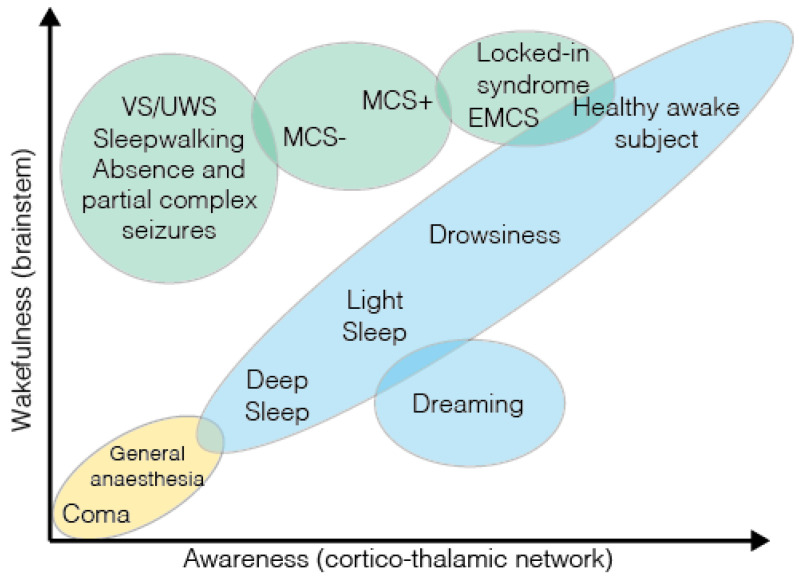
**Clinical scales of consciousness.** A clustering of disorders of consciousness in humans represented on scales of awareness and wakefulness. Adapted from [2]. In neurophysiological recordings, signatures of awareness have been found in cortico-thalamic activity, whereas wakefulness corresponds to activity in the brainstem and associated systems [1,2]. Abbreviated legends: VS/UWS (vegetative state/unresponsive wakefulness state) [21]; MCS(+/−) (minimally conscious state plus/minus), EMCS (emergence from minimally conscious state) [22].

In patients with disorders of consciousness ranging from coma, locked-in syndrome to those in vegetative states, levels of consciousness are assessed through a battery of behavioral tests as well as physiological recordings. Cognitive awareness in patients is assessed by testing cognitive functions using behavioral and neurophysiological (fMRI or EEG) protocols [1]. Assessments of wakefulness/arousal in patients are based on metabolic markers (in cases where reporting is not possible) such as glucose uptake in the brain, captured using PET scans. More generally, in [1,2], awareness and wakefulness have been proposed as a two-dimensional operational definition of clinical consciousness, shown in Figure 1 below. While awareness concerns higher and lower-order cognitive functions enabling complex behavior; wakefulness results from biochemical homeostatic mechanisms regulating survival drives and is clinically measured in terms of glucose metabolism in the brain. In fact, in all known organic life forms, biochemical arousal is a necessary precursor supporting the hardware necessary for cognition. In turn, evolution has shaped brains in such a way so as to support the organism’s basic survival (using wakefulness/arousal) as well as higher-order drives (using awareness) associated to cooperation and competition in a multi-agent world [20]. Awareness and wakefulness thus taken together, form the clinical markers of consciousness.

The clinical definition (or criterion) of consciousness enables a practical classification of closely associated states and disorders of consciousness into clusters on a bivariate scale with awareness and wakefulness on orthogonal axes. Under healthy conditions, these two levels are almost linearly correlated, as in conscious wakefulness (high arousal and high awareness) or in deep sleep (low arousal and low awareness). However, in pathological states, wakefulness without awareness can be observed in the vegetative state [1], while transiently reduced awareness is observed following seizures [23]. Patients in the minimally conscious state show intermittent and limited non-reflexive and purposeful behavior [24,25], whereas patients with hemispatial neglect display reduced awareness of stimuli contralateral to the side where brain damage has occurred [26].

The question is, how can one extrapolate wakefulness and awareness for non-biological systems in order to obtain homologous scales of consciousness that can be applied to artificial systems? As noted above, wakefulness results from autonomous homeostatic mechanisms necessary for the self-preservation of an organism’s germ line in a given environment. In other words, it is tied to self-sustaining life processes necessary for basic survival, whereas awareness refers to functionalities pertaining to estimating or predicting states of the world and optimizing the agent’s own actions with respect to those states. If biological consciousness as we know it, is supported via a synergistic interaction between metabolic and cognitive processes, then how should this insight be extended to conceive a functional notion of consciousness in synthetic systems? One way of doing so might be generalizing from scales of wakefulness to those of generic autonomic processes; likewise, generalizing from scales of awareness to those of generic computational processes. As such, most autonomic processes are usually considered to be running below the radar of consciousness (or unconscious in certain usages). On the other hand, computational processes provide for mechanistic descriptions for many neural and cognitive functions associated to consciousness. However, as evident from the examples above (including those of disorders of consciousness), biological forms of consciousness seem to require both types of processes. For that reason, we will consider both, autonomic and computational processes when formulating homologous scales of consciousness in synthetic systems.

### 2.2. Candidate Measures in Brain and Behavioral Studies

Given the above discussion of clinical scales of consciousness (based on wakefulness and awareness), in Section 5, we will attempt to identify the homologues of these biological scales for artificial systems. As a precursor to that discussion, in this subsection we will review prominent candidate measures of autonomy and computation in brain and behavioral sciences.

Measures of autonomy and computation, including information processing performed by cognitive agents, have been discussed in various psychometric [27] and neurophysiological studies [28]. However, generalizing these measures to artificial systems and comparing those values to biological systems is certainly not so straightforward (due to completely different processing substrates as well as differing comparative benchmarks). Nonetheless, biological/cognitive measures of autonomy and computation suggest a first step in this direction. For example, [27] introduced an “Index of Autonomous Functioning”, tested on healthy human subjects (via psychometric questionnaires). This index aims to assess psychological ownership, interest-taking and susceptibility to external controls. This is similar to the concept of volition (or agency), introduced in the cognitive neurosciences [29,30], which seeks to determine the neural correlates of self-regulation, referring to actions regulated by internal drives rather than exclusively driven by external contingencies.

Psychometric attempts to quantify awareness have been discussed in [31] in the context of a unified psychological theory of self-functioning. In consciousness research, a measure of awareness that has gained a lot of traction is integrated information [32] (often denoted as Φ). This is an information-theoretic complexity measure. It was first introduced as a measure that might be useful for realistic neural data. Based on mutual information, Φ has been touted as a correlate of consciousness [32]. Integrated information is loosely defined as the quantity of information generated by a network as a whole, due to its causal dynamical interactions, over and above the information generated independently by the disjoint sum of its parts. As a complexity measure, Φ seeks to operationalize the intuition that complexity arises from simultaneous integration and differentiation of the network’s structure and dynamics, thus enabling the emergence of the system’s collective states. The interplay between integration and differentiation generates information that is highly diversified yet integrated, creating patterns of high complexity. Following initial proposals [32,33,34], several approaches have been developed to compute integrated information [35,36,37,38,39,40,41,42,43,44,45,46,47,48,49,50].

Notably the measure discussed in [38] will be relevant for our discussion as it develops methods for large-scale network computations of integrated information, applied to the human brain’s connectome network. The human connectome network consists of structural connectivity of white matter fiber tracts in the cerebral cortex, extracted using diffusion spectrum imaging and tractography [51,52] (see [53,54,55,56,57,58] for visualization of neurodynamical data and model dynamics on this network). Compared to a randomly re-wired network, it was seen that the particular topology of the human brain generates greater information complexity for all allowed couplings associated to the network’s attractor states, as well as its non-stationary dynamical states [38].

Φ as described above, is not specific to biological systems and can also be applied to artificial dynamical systems. In Section 5 we will exploit the applicability of Φ and use it as a generic measure of computational complexity for artificial systems.

## 3. Synthetic Consciousness? Insights from Synthetic Biology and Artificial Intelligence

Let us now look at the evidence in synthetic biology and AI to see how these systems qualitatively compare to biological systems. Oftentimes, our methods for probing biological systems can be limited due to natural design constraints. On the other hand, the potential for exploring synthetic counterparts provides a unique opportunity to probe the nature of life and intelligence processes. It has been suggested that artificial simulations, in silico implementations and engineered alternatives are in fact, much needed for understanding the origins of evolutionary dynamics, including cognitive transitions [59]. What can be learned in relation to consciousness from artificial agents?

Within the context of non-cognitive phenomena, synthetic biology provides a valuable example of the classes of relevant questions that can be answered. Examples are the possibility of creating living systems from non-living chemistry, generating multicellular assemblies, creating synthetic organoids or even artificial immune systems. Here advanced genetic engineering techniques along with a systems view of biology has been able to move beyond standard design principles provided by evolution. Examples of this are new genetic codes with extra genetic letters in the alphabet that have been designed and successfully inherited [60], synthetic protocells with replicative potential [61] and even whole synthetic chromosomes that have defined a novel bacterium species [62]. Ongoing research has also revealed the potential for creating cognitive networks of interacting microorganisms capable of displaying collective intelligence [63].

Of course, the criteria for consciousness, as stated in sections above, are not even remotely satisfied by any of these synthetic systems. They either have some limited form of intelligence or life but not yet both. Nevertheless, there have been some noteworthy recent developments in these areas. In this context, AlphaGo’s 2016 feat in beating the top human Go champion was remarkable for a couple of reasons. Unlike Chess, possible combinations in Go run into the millions and when played using a timer, any brute-force algorithm trying to scan the entire search space would simply run out of computational capacity or time. Hence, an efficient pattern recognition algorithm was crucial to the development of AlphaGo, where using deep reinforcement learning, the system was trained on a large number of games [64]. Most interestingly, it played counterintuitive moves that shocked the best human players and the sole game of the series that Lee Sedol, the human champion, won out of five, was only possible after he himself adopted a brilliant counterintuitive strategy. Subsequent AI systems such as AlphaGo Zero, AlphaZero and MuZero have gone even further. While AlphaGo learnt the game by playing thousands of matches with amateur and professional players, AlphaGo Zero learnt by playing against itself over and over again, starting from completely random play, while reinforcing successful sequence of plays through the weights of its deep neural networks [64,65]. This aspect of playing itself is akin to training via social interactions as will be described below. Then we have AlphaZero, which is a single system that taught itself from scratch how to master the games of chess, shogi and Go [66]. And MuZero takes these ideas one step further. It matches the performance of AlphaZero on Go, chess and shogi, while also mastering a range of visually complex Atari games, all without being told the rules of any game [67].

Thus, AI such as AlphaGo and its successors do demonstrate a rather broad form of domain intelligence (that is within a game or across games). In contrast, most forms of biological problem-solving capabilities span across domains (related to ecologically-realistic constraints). Moreover, one would agree that AlphaGo is not equipped with any form of wakefulness mechanisms coupled to its computational capabilities [68].

The same can be said for other state-of-the-art AI systems including deep convolutional neural networks, or deep recurrent networks. Both these latter architectures were inspired from Hubel and Weisel’s groundbreaking work on the coding properties of the visual system, which led to the realization of a hierarchical processing architecture [69]. Today deep convolutional networks are widely used for image classification [70] and recurrent neural networks for speech recognition [71], among countless other applications. Recent developments have advanced this by virtue of larger data sets and more computational power. For example, there have been attempts to build biologically-plausible models of learning in the visual cortex using recurrent neural networks [72]. In summary, deep architectures have made remarkable progress in domain-specific AI.

However, asking whether AI can be conscious in exactly the same way that a human is, is similar to asking whether a submarine can swim. Even if it did so, it might well do so differently. If the goal of a system is to learn and solve complex tasks close to human performance or better, current machines are already doing that in specific domains [73,74,75,76,77,78,79]. However, these machines are still far from learning and solving problems in generic domains and more importantly, in ways that would couple its problem-solving capabilities to its autonomous survival drives. On the other end of things, neither have any of the synthetic life systems discussed above been used to build architectures with complex computing or cognitive capabilities. Nevertheless, this does suggest that a future synthesis between artificial life forms and AI could be evaluated using homologous scales of consciousness to the ones currently applicable to biological beings. This plausible form of synthetic consciousness, if based on a life form with different survival drives and mechanisms, along with non-human forms of intelligence or computation, would also likely lead to non-human behavioral outcomes.

In summary, these phenomenological considerations suggest that autonomic and computational complexity provide the necessary abstractions to wakefulness and awareness, which can be applied to a wide spectrum of synthetic agents in terms of their underlying mechanistic processes. In the next section, we will make the case for a third kind of complexity, necessary to build the morphospace of consciousness, namely, social complexity.

## 4. The Function of Consciousness: Insights from Evolutionary Game Theory and Cognitive Robotics

Reviewing insights from evolution and cognitive robotics, this section looks at the functional role of consciousness [20,80,81,82,83]. The biological substrates of consciousness presumably evolved through natural selection driven by social co-operation and competition. This can be framed in the context of evolutionary game-theory. In [80,84] it was suggested that rather than being thought of as a problem, consciousness could rather be seen as a solution to the problem of autonomous goal-oriented action, when faced with a world filled with other agents. This was formulated as the *H5W problem*.

### 4.1. The H5W Problem

What does an agent operating in a social world need to do in order to optimize its fitness? It needs to perceive the world, to act, and through time, to understand the consequences of its actions so it can start to reason about its goals and how to achieve them. This requires building a representation of the world grounded on the agent’s own sensorimotor history and use that to reason and act. The agent will witness a scene of agents, including itself, and objects interacting in various manners, times and places. This comprises the six fundamental problems that the agent is faced with, together referred to as the H5W problem [80,84]: In order to act in the physical world an agent needs to determine a behavioral procedure to achieve a goal state; that is, it has to answer the HOW of action. In turn this requires the agent to: (a) Define the motivation for action in terms of its needs, drives and goals, that is, the WHY of action; (b) Determine knowledge of objects it needs to act upon and their affordances in the world, pertaining to the above goals, that is, the WHAT of action; (c) Determine the location of these objects, the spatial configuration of the task domain and the location of the self, that is, the WHERE of action; (d) Determine the sequencing and timing of action relative to dynamics of the world and self, that is, the WHEN of action; and (e) Estimate hidden mental states of other agents when action requires cooperation or competition, that is, the WHO of action.

While the first four of the above questions suffice for generating simple goal-oriented behaviors, the last of the Ws (the WHO) is of particular significance as it involves intentionality, in the sense of estimating the future course of action of other agents based on their social behaviors and psychological states. However, because mental states of other agents that are predictive of their actions are hidden, these can at best be inferred from incomplete sensory data such as location, posture, vocalization, social salience, etc. As a result the acting agent faces the challenge to univocally assess, in a deluge of sensory data those exteroceptive and interoceptive states that are relevant to ongoing and future action and therefore has to deal with the ensuing credit assignment problem in order to optimize its own actions. Furthermore, this results in a reciprocity of behavioral dynamics, where the agent is now acting on a social and dynamical world that is in turn acting upon itself. It was proposed in [84] that consciousness is associated to the ability of an agent to maintain a transient and autonomous memory of the virtualized agent-environment interaction, that captures the hidden states of the external world, in particular, the intentional states of other agents and the norms that they implicitly convey through their actions.

### 4.2. Evolutionary Game Theory

From the above, we surmise that the function of consciousness is to enable an acting agent to solve its H5W problem while being engaged in social cooperation and competition with other agents in its evironment, who are trying to solve their own H5W problem in a world with limited resources [80,81]. This brings our discussion to the setting of evolutionary game theory.

First, consider a scenario with only a small number of other agents. Then any given agent might use statistical learning approaches to learn and classify behaviors of the few others agents in that game. For example, multiple robots interacting to learn naming conventions of perceptual aspects of the world [85]. At the least, this requires embodiment so that agents can interpret perceptual cues presented by other agents (for example, by pointing at objects) [86]. Another example is the emergence of signaling languages in sender-receiver games based on replicator dynamics described by David Lewis in 1969 in his seminal work, Convention [87,88]. These are all examples where social norms are acquired in the process of iterative multi-agent interactions, and can thus be investigated in the setting of multi-agent game theory using evolutionary algorithms.

Note however, that most game-theoretic strategies involving statistical learning are computationally feasible only when a limited number of players are involved. They are often sub-optimal in the event of an explosion in the number of players (see [89] for an overview of these limits). Likewise, in a social environment comprising a large number of agents trying to solve the H5W problem, machine learning strategies for reward and punishment valuations may soon become computationally unfeasible for an agent’s processing capacities and memory storage. Therefore, for a large population to sustain itself in an evolutionary game involving complex forms of cooperation and competition would require strategies other than merely data-driven statistical learning. One such strategy involves modeling and inferring intentional states of the self and that of other agents. Emotion-driven flight or fight responses depend on such intentional inferences and so do higher-order psychological drives. The mechanisms of consciousness enable such strategies, whereas, contemporary AI systems such as AlphaGo do not possess such capabilities.

The importance of the role that sociality plays in surviving a multi-agent world suggests a possible function of consciousness: it is a mechanism that enables agents to learn and acquire complex social game-theoretic strategies based on emotional cues. From an evolutionary perspective, social behaviors result from generations of cooperation–competition games, with natural selection filtering out unfavorable strategies. Presumably, winning strategies were eventually encoded as anatomical mechanisms, such as emotional responses. The complexity of these behaviors depends on the ability of an agent to make complex social inferences.

While evolutionary game theory itself does not hinge on consciousness (and there are plenty of examples of emergent behaviors acquired in iterative multi-agent games involving reciprocating agents driven by cultural cues, where the presence or need for consciousness does not arise [90]), nor is consciousness the end-product of all evolutionary games; the key point we wish to emphasize here is that the mechanisms of biological consciousness, which allow organisms to have highly flexible autonomous action and cognitive processing capabilities, provide a competitive advantage to agents operating in complex social environments. Based on fossil records, the evolutionary and genetic origins of consciousness have been traced back to the Cambrian Period over 500 million years ago [91], when early vertebrates with somatotopically-organized neural representations acquired sensory capabilities (with vision being postulated as the first conscious sense [91]). These early markers of sensory consciousness enabled agents to navigate complex social scenarios (without having to rely exclusively on extensive computational resources).

**Figure 2:**
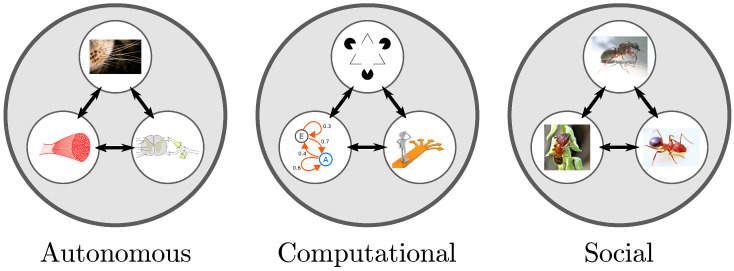
**Schematic representation of autonomic, computational and social complexity.** Each complexity measure is illustrated as a whole (the large circles) constituted of its parts (the inner circles), their interactions (the arrows) and the emerging properties resulting from these interactions (the inner space within the large circles, in light grey). Autonomic complexity (left) refers to the collective phenomena resulting from the interactions between typical components of reactive behavior such as sensors (illustrated by whiskers in the top inner circle), actuators (illustrated by a muscle in the bottom-left inner circle) and low-level sensorimotor coupling (illustrated by a spinal cord in the bottom-right inner circle). Computational complexity is associated to higher-level cognitive processes such as visual perception (top inner circle), planning (bottom-left inner circle) or decision making (bottom-right inner circle). Social complexity is associated to interactions between individuals of a population, such as a queen ant (top inner circle), a worker ant (bottom-left inner circle) and a soldier ant (bottom-right inner circle).

**Figure 3:**
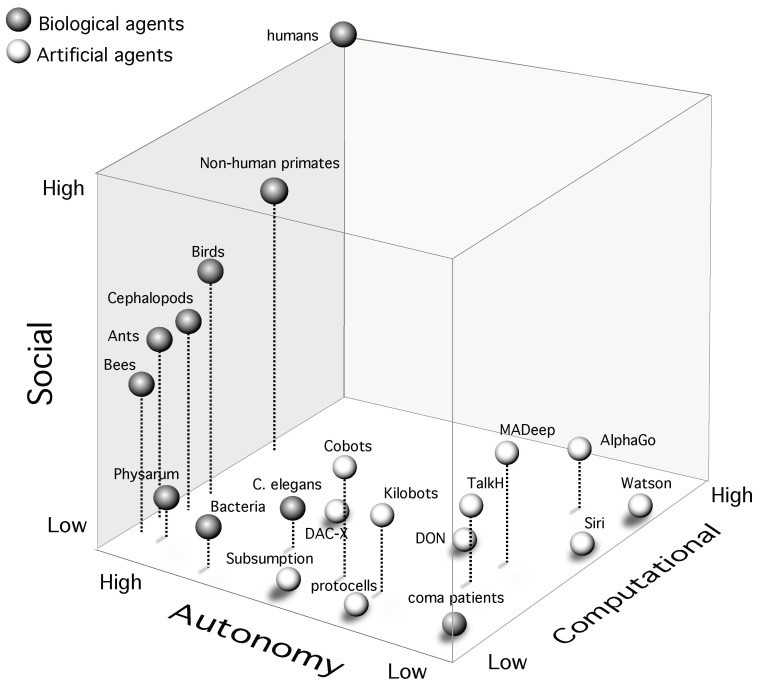
**Morphospace of consciousness.** Autonomous, computational and social complexity constitute the three axes of the consciousness morphospace. Human consciousness is used as a reference in one corner of the space. Current AI implementations cluster together in the high computation, low autonomy and low social complexity regime, while multi-agent cognitive robotics cluster around low computational, but moderate autonomous and social complexities. Abbreviated legends: MADeep (multi-agent deep reinforcement system) [120]; TalkH (talking heads) [86]; DQN (deep Q-learning) [114]; DAC-X (distributed adaptive control) [121], CoBot (cockroach robot) [122], Kilobot (swarm robot) [119], Subsumption (mobile robot architecture) [123].

All of this discussion suggests a third dimension in the morphospace of consciousness (see Figure 2 and Figure 3 below), namely, social complexity, which serves as a measure of an agent’s social intelligence.

## 5. Three Kinds of Complexity to Characterize Consciousness

In this section we discuss the construction of the aforementioned morphospace as well as candidate complexity measures to parametrize it.

### 5.1. Why Distinguish Between Complexity?

The phenomenology of consciousness draws upon a variety of empirical disciplines including cognitive and clinical neuroscience, synthetic biology, artificial intelligence, evolutionary biology and robotics. The theoretical challenge is then to find a formal explanatory framework that provides an abstraction of the phenomenology across substrates. One attempt at doing so has been through complexity measures. However, based on the evidence discussed above, a universal complexity measure may be insufficient to parse through the types of process and functional specifications supporting consciousness. This point has also been mentioned in [92], albeit from purely clinical considerations. Hence, in this work, we make the case for at least three kinds of complexity, based on process types. These are autonomic complexity, computational complexity and social complexity (see Figure 2 and Table 1 below). Table 1 lists the respective building blocks, systems-level realizations, and associated emergent phenomena for each of these complexity kinds.

A common type of complexity measure, that is often discussed in neuroscientific and consciousness-related paradigms, is a whole-versus-parts measure. Here, a system’s complexity C is defined by how much an integrated whole outdoes the sum of its independent parts in terms of an information processing metric. Generally, $C=I_{substrate}-\sum_{\left\{parts\right\}}I_{part}$, where I refers to an appropriate type of information. For instance, when I is the conditional entropy, C yields the measure Φ of integrated information theory and its many derivatives. Besides whole-versus-parts measures, there are a host of others which capture different aspects of information processing in complex systems. Below, we discuss the relevance of each of these measures with respect to the three kinds of complexity proposed in this article and how they may be collectively used for labelling states of consciousness.

Autonomic complexity $C_{Autonomic}$ measures the complexity of processes enabling the system to act autonomously in its environment. In eukaryotes, autonomic action refers to arousal mechanisms resulting from coordinated nervous system activity; in prokaryotes, this refers to reactive behaviors such as chemotaxis, stress responses to temperature, toxins, mechanical damage, etc., all of these resulting from coordinated cellular signaling processes; in robotics, autonomic systems refer to homeostatic mechanisms driving reactive behaviors. Hence, autonomic complexity quantifies information processing by the collective dynamics of the systems driving autonomous behaviors.

On the other hand, computational complexity $C_{Computational}$ refers to the ability of an agent to integrate information over space and time across computational or cognitive tasks. In higher biological forms, this complexity is typically associated to neural processes; in artificial computational systems, it refers to microprocessor signaling. The distinction between $C_{Computational}$ and $C_{Autonomic}$ is specified by the tasks that they refer to, rather than substrates. $C_{Autonomic}$ refers to autonomous control loops, whereas $C_{Computational}$ refers to computational and inferential mechanisms.

In whole-versus-parts terminology, social complexity $C_{Social}$ would refer to information generated by a population as a whole, during the course of social interactions, over the information generated additively by individual agents of that population. Unlike $C_{Autonomic}$ or $C_{Computational}$, $C_{Social}$ is not assigned to an individual, but rather to a specific population (its own species) within which the individual has been interacting. As discussed earlier, by way of social games, these interactions are believed to have contributed to the emergence of the agent’s consciousness on an evolutionary time-scale. Note that $C_{Social}$ as defined here, does not refer to group consciousness (we will discuss that in following sections), rather it quantifies the environmental complexity due to a population of agents (this in turn, applies selection pressures on individual agents).

### 5.2. Candidate Complexity Measures

This section takes an overview on candidate complexity measures for quantifying each of the three complexity kinds discussed above. Table 2 below provides a summary of these measures.

Let us begin with autonomic complexity. Besides integrated information, which may also be customized to the components of the autonomic system, other measures that have proven more practical for capturing the complexity of autonomous processes in systems are morphological computation [93,94], synergistic information [95,96] and the index of autonomous functioning [27,97,98]. The first of these is particularly useful for systems with high morphological dexterity such as in biology and soft robotics. It captures the extent to which a system’s morphological properties are used to delegate and distribute its informational processing capabilities towards the goal of autonomous action. Synergistic information refers to information provided by the simultaneous knowledge of multiple variables, that is not available from any of the individual variables by themselves. In [96], this was used to show how an agent’s cue sensors jointly carry cue information with the agent’s interneurons (in fact, in this example, this measure quantifies the coupling between systems referring to $C_{Autonomic}$ and $C_{Computational}$). The index of autonomous functioning has extensively been used in human behavioral studies to quantify regulation of action by the self. All these studies, particularly [98] emphasize the necessity of autonomy (and hence, autonomic complexity) for systems realizing goal-oriented behaviors.

Now let us turn our attention to measures capturing systems and processes referring to computational complexity $C_{Computational}$. These have been studied extensively in neuroscience and AI. In the context of consciousness research, the most prominent among these is the measure of integrated information, Φ. However, there have been several candidates for this measure and its many approximations. The earliest version of IIT was based on a measure called neural density [32] (see also [33,34]). Subsequently, version 2 of the theory, IIT v2, defined Φ in terms of a Kullback-Leibler divergence, which was used as a relative entropy measure to quantify the information generated by the whole over the sum of its parts [43,99]. The current version of the theory, dubbed IIT 3.0, uses the Earth Mover’s Distance (EMD) [46]. Despite its conceptual appeal, the algorithm proposed by IIT has been computationally intractable for realistic biological or artificial systems. This is where either related or approximate integrated information measures have been useful. Examples of related measures include stochastic interaction (also called total information flow) [49], stochastic integrated information [35,38,44] and geometric measures of integrated information [42]. Examples of various empirical approximations to Φ include the ‘Perturbational Complexity Index’ based on Lempel-Ziv compression [100] and causal connectivity based on Granger causality [101] (see [40] for a review of theoretical and empirical consciousness measures).

Besides integrated information, other information-theoretic measures that have been used in cognitive and computational neuroscience are mutual information and specific information, both of which have been used in neural coding paradigms [96]. From partial information decomposition methods, one has measures of redundant information, unique information and synergistic information [95,96]. These measures are relevant in situations where multiple sources potentially carry information about a measurement outcome or cue variable. Synergistic information refers to the property of multiple random variables cooperating to predict, or reduce the uncertainty of, a single target variable. In general cases, these are quite difficult to compute. In the case of two and three source variables, a formulation of these measures can be found in [95,96]. Yet another class of information-theoretic measures applicable to computational systems is that which describes the dynamics of information processing. Examples of these information-theoretic measures are transfer entropy, information gain and information transfer (discussed in [96]). The last two of these are of practical relevance. They have been tested on an artificial cognitive agent with a brain, body and environment [96]. This study shows that information-theoretic analysis reveals important insights on how task-relevant information flows through the embodied agent and is combined into a categorization decision. Furthermore, a dynamical systems analysis reveals the key geometrical and temporal interrelationships underlying the above categorization task performed by the agent.

Finally, let us discuss social complexity. There are two broad measures that have been discussed in the literature for this kind of complexity. One is, not surprisingly, integrated information, discussed in [102]. The other is the collective intelligence factor, discussed in [103,104], which refers to how well groups perform on a diverse set of group problem-solving tasks. The primary influences on a group’s collective intelligence were identified to as the following: (a) the group composition (e.g., the members’ skills, diversity, and intelligence) and (b) the group interaction (e.g., structures, processes, and norms of the group). Other related works that discuss collective intelligence include [105,106,107,108]. All of these cited measures seek to capture social complexity of groups with respect to complex tasks involving the group as a whole. Below we shall see that this kind of complexity is important for the morphospace of consciousness where the groups refer to species of animals or kinds of technologies.

### 5.3. Constructing the Morphospace

Using these definitions for the three complexity kinds, we construct the morphospace of consciousness in Figure 3. While this space is only a first attempt at constructing a common framework for biological and artificial agents, the precise coordinates of various systems within this morphospace might change due to the rapid pace of new and developing technologies, but we expect the relative locations of each example to remain the same. We start with the human brain, which is taken as the benchmark in this space, defining a limit case located at one upper corner with highest scores on all the three axes. The human brain can perform computational tasks across a variety of domains such as making logical inference, planning an optimal path in a complex environment or dealing with recursive problems and hence leads with respect to computational complexity due to these cross-domain capabilities. On the social axis, human social interactions have resulted in the emergence of language, music, art, culture or socio-political systems. Other biological entities such as non-human primates [109,110] or social insects would score lower on the social and computational axis than humans. Additionally, other species of vertebrates such as some types of birds and cephalopods have been shown to exhibit complex behavior and possess sophisticated nervous systems. These two groups have actually been enormously useful in the search for animal consciousness [111,112].

Current AI systems such as IBM Watson [113], AlphaGo [64], DQNs [114] and Siri [115] are powerful computing systems over a narrow set of domains, but in their current form they do not show general-purpose functionality, that is, the capacity to independently interact with the world and successfully perform multiple tasks in different domains [116], or as proposed by Allen Newell, the capability with which anything can become a task [117]. These AI systems are still clustered high on the computational axis, but lower than humans (due to their domain-specificity). Also they score low on autonomic and social complexity. Synthetic forms of life such as protocells show some levels of autonomous functioning, reacting to chemicals and stressors, but currently show minimal capabilities for computation or inference and minimal interactions with other agents [118].

Interest in the field of multi-agent robotics has led to the rise of machines where emergent collective behaviors, e.g., coordination (KiloBot [119], Multi-Agent Deep Network [120]) or shared semantic conventions (Talking Heads [86]) self-organize out of multi-agent interactions. These systems are designed to display simple forms of navigation, object-detection, etc., while interacting with other agents performing the same task. However, they show lower social and autonomic complexity than most biological agents. Being embodied systems, they currently score lower on computational complexity than heavy-powered AI systems such as Watson or AlphaGo.

An important use of the morphospace within evolutionary biology is related to the actual occupation of this space by different solutions. Notice that in the morphospace in Figure 3, a large part of the space is left vacant. A similar observation was made in [5] in the context of the morphospace of synthetic organs and organoids. In both cases, such an observation points towards new classes of artificial life and intelligence. Most present-day artificial systems (both, synthetic biology and AI), depicted in the morphospace, remain in the lower part of the cube. This is indicative of the currently minimal role played by social context in the development of these systems. On the other hand, in natural systems, social interactions have played an important role in shaping the minds of the organisms (those close to the left wall in Figure 3, involving high autonomy and sociality). Complex organisms equipped with brains and exhibiting cooperative behavior have evolved to live together with others. This is because social synergies increase the resilience of the group to many environmental and predatory challenges.

### 5.4. Relation to General Intelligence

How do our discussions on consciousness relate to theories of general intelligence? The idea that consciousness resides in select regions of a morphospace, that is constructed from function-specific types of complexity, has implications for any theory of general intelligence. The dimensions of our morphospace implicitly entail (or rather subsume) distinct types of intelligence. In cognitive psychology, manifestations of human intelligence have been discussed in the context of Howard Gardner’s theory of multiple intelligences [124]. Here we want to understand how the dimensions of our morphospace help group different types of intelligences. This works as follows (We thank Carlos E. Perez for bringing this point to our attention). A discussion about how Gardner’s intelligence types may be realized in machines using deep learning can be found in his recent book [125]). The autonomic axis reflects adaptive intelligence found in biological organisms. This encapsulates Gardner’s kinesthetic, musical and spatial intelligence (some of these also require computational complexity). The computational axis refers to recognition, planning and decision-making capabilities that we find in computers as well as in humans. These are tasks involving logical deduction or inference. Hence, this complexity refers to those types of intelligences that require computational capabilities, such as logical reasoning, linguistic intelligence, etc. The third axis of the morphospace, social complexity, relates to social capabilities required for interacting with other agents. This refers to interpersonal and introspective intelligence, in Gardner’s terms. These types of intelligences are also associated to the evolution of language, social conventions and culture. Then there are also other types of intelligences described in Gardner’s theory such as naturalistic and pedagogical intelligence, which involve a composition of social and computational complexity.

As described above, the defining dimensions of our morphospace account for all of the multiple types of intelligence proposed by Gardner. Taking these intelligence (or their associated complexity) types into account, while building artificially intelligent machines, elucidates the wide spectrum of problems that future AI could potentially address. In the light of both, Gardner’s theory and Newell’s criteria, our morphospace, in fact, suggests that consciousness as we know it, subsumes a specific form of integrated multiple intelligence. Note that one ought to be careful to *not* claim that consciousness ’*is*’ general intelligence. Following William James, in cognitive psychology, consciousness is seen as a process that enables action for survival purposes [126]. We claim that this process, enabling action, constitutes mechanisms and phenomenology that realize an integration of specific types of intelligences and their associated complexities in such a way so as to meet survival goals. On the other hand, intelligence by itself can be thought of as any task-specific capability (or a process realizing that capability), that is not necessarily tied to existential pressures [127]. However, currently we have yet to fully understand how several of the intelligence types mentioned above, especially the non-computational ones [128], can be functionally realized in machines, let alone understanding the mechanisms that lead to integration of types. Nonetheless, given the myriad of recent advances in human-machine interactions, a complexity-based conceptualization of consciousness provides a practical and quantitative framework for studying ways in which interactions with machines might enhance our joint complexities and competences.

The outlook of these complexity kinds with respect to general intelligence is that systems and processes referring to computational intelligence will bring about new cures in medicine, new scientific understanding, and more efficient and less wasteful processes. Machines with autonomous intelligence capabilities will bring about greater conveniences such as self-driving automobiles, robotic care-takers in the workplace and in the home, and intuitive user interfaces. The third kind, systems with social intelligence, will be beneficial with regards to advertising to the masses, promoting global causes and managing social unrest.

### 5.5. Other Embodiments of Consciousness in the Morphospace

What other forms of consciousness does our morphospace suggest? Because there is no precise definition or consensus to benchmark consciousness even in biological life forms, the best one can do at the moment is to pursue a comparative functional approach as has been followed here. The three complexity axes on the morphospace encapsulate processes necessary to support functions that consciousness serves. Moreover, from the earlier discussion on multiple intelligence types being distributed across the morphospace, one may ask what forms of systems might reside in distinct regions of this space. Higher biological life-forms, those which are generally believed to possess some degree consciousness (in terms of reportable behaviors), tend to cluster closer to one corner of the cube in Figure 3. The other corners are suggestive of agents or systems with different embodiments and functionalities. Below we identify these embodiments and the form of consciousness or intelligence that they might potentially refer to (again, purely on functional grounds).

**Figure 4:**
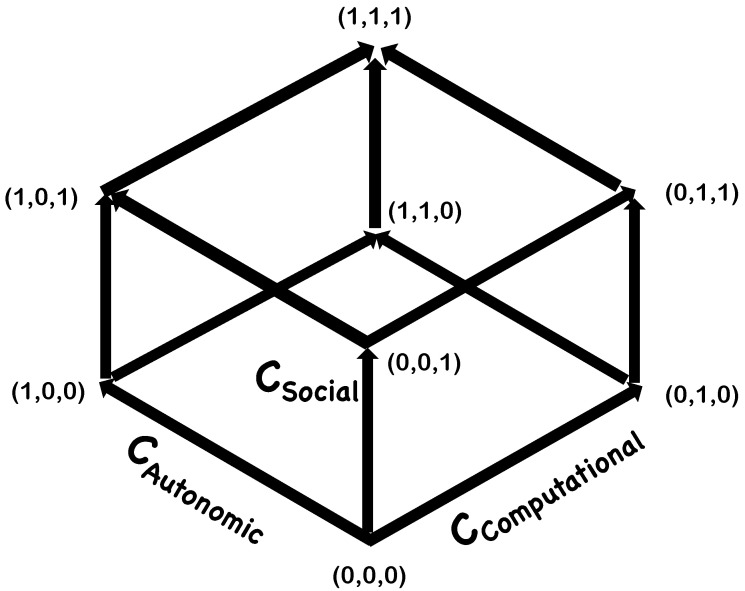
**A Boolean Graphical** **Representation of the Morphospace.**

To illustrate these embodiments, it is instructive to represent the morphospace as a Boolean graph, where vertices are labelled by their corresponding cartesian co-ordinates in the cubic morphospace and edges refer to one of the three complexity axes along the cube (Figure 4). The (1,1,1) vertex corresponds to human consciousness. The (1,0,0) vertex and (0,1,0) vertex correspond to present day synthetic biological systems and AI technologies, respectively. Neither of them are considered conscious. Examples of current technologies near the (0,0,1) vertex would be highly interactive reactive systems. Even these are not what one would consider conscious. The (1,1,0) vertex corresponds to an agent that is highly autonomous and computational, but lacking social drives. Evolutionarily, such agents would be disfavored. Technologically, they offer somewhat similar utilities as agents on either the (1,0,0) or (0,1,0) vertex. For our purposes, the interesting vertices are (1,0,1) and (0,1,1). This is where future intelligent technologies or potentially new forms of conscious systems may be found. Below we identify three system embodiments, corresponding to potential forms of consciousness, that occupy these vertices.

#### 5.5.1. Synthetic Consciousness

In Section 3 above, we alluded to two kinds of synthetic systems: synthetic biological systems and AI technologies. The question is, what augmentations would these systems need in order to be able to solve the H5W problem (of Section 4), with respect to their conspecifics? When that criterion is met, these systems could be considered to possess basic forms of consciousness on functional grounds. For certain, this would require them to have sufficient social complexity. Systems located near the (1,0,1) vertex would show behaviors similar to some biological life-forms. Systems located near the (0,1,1) vertex would be where current efforts in AGI (artificial general intelligence) are trying to get. Of course, at present, we do not know of any objective tests to ascertain if these systems on the (1,0,1) and (0,1,1) vertices may have first-person experiences (this criticism holds also for many biological species that one would otherwise argue as possessing phenomenological traits of consciousness). The comparison of these to a form of consciousness is made on behavioral grounds. Alternatively, one might as well refer to these systems as a form of synthetic general intelligence.

#### 5.5.2. Group Consciousness

In a sense, biological consciousness itself can be thought of as a collective phenomenon where individual cells making up an organism are themselves not considered to be conscious (with respect to the three complexity measures defining the morphospace), even though the organism as a whole is. But what happens when the system itself is not localized? We postulate group consciousness as an extension of the above idea to composite or distributed systems that display levels of computational, autonomic and social complexity that are sufficient to answer the H5W problem. Note that, as per this specification of group consciousness, the group itself is treated as one entity. Hence, social complexity now refers to the interactions of this group with other similar groups.

This bears some resemblance to the notion of collective intelligence, which is a widely studied phenomenon in complex systems ranging from ant colonies [107], to a swarm of robots (the Kilobot in [119] and the CoRobot in [122]), to social networks [129]. However, these are usually not thought of as conscious systems. As a whole, they are not considered as autonomic forms with survival drives that compete or cooperate with other similar agents. However, these distinctions begin to get blurred during transient epochs when collective survival comes under threat. For example, when a bee colony comes under attack by hornets, collectively it demonstrates a prototypical survival drive, similar to lower-order organisms. Other examples of such behaviors have also been studied in the context of group interactions in humans, where social sensitivity, cooperation and diversity have been shown to correlate with the collective intelligence of the group [103]. Following this, the notion of collective intentionality has been discussed in [108]. More recently, [102] have applied integrated information Φ to group interactions, suggesting a new kind of group consciousness. While it is known that Φ in adapting agents increases with fitness [130], one can ask a similar question for an entire group: what processes (evolutionary games, learning, etc.) enable an increase in all three complexity types for an entire group such that it can solve the H5W problem while cooperating or competing with other groups?

For these reasons, this type of system, if conscious, in terms of being able to solve the H5W problem with respect to its conspecifics (other groups), will cluster around the (1,0,1) vertex of the morphospace.

#### 5.5.3. Simulated Consciousness

Our discussions on complexity kinds also suggest yet another potential type of consciousness, namely, simulated consciousness, wherein embodied virtual agents in a simulated reality interact with other virtual agents, while satisfying the complexity bounds that enable them to answer the H5W questions within the simulation. In this case, consciousness is strictly confined to the simulated environment. The agents cannot perceive or communicate with entities outside of the simulation but satisfy all the criteria we have discussed above within the simulation. How these embodied virtual agents could acquire consciousness is not yet known. Presumably by evolving across multiple generations of agents that adapt and learn to optimize fitness conditions. It is also not clear what precise traits or mechanisms would have to be coded into the simulation (as initializations or priors) in order to enable consciousness to evolve. The point here is simply that the same criteria that we have identified with consciousness in biological agents in the physical world, could in principle be admitted by agents within a simulation and confined to their interactions within that simulation. This has parallels to the notion of “Machine Consciousness” discussed in [131], which proposes that neural processes leading to consciousness might be realizable as a machine simulation (it even goes further to claim that computer systems might someday be able to emulate consciousness). At the moment, these are all open challenges in AI and consciousness research. Examples of studies discussing embodied virtual agents can be found in the work of [132,133]. More recent implementations of embodied virtual agents have been using gaming technology, such as the Minecraft platform [134,135].

Simulations, if conscious in the functional sense—that is, being able to solve the H5W problem with respect to its conspecifics (other groups) within the simulation—will cluster around the (0,1,1) vertex of the morphospace.

## 6. Discussion

The objective of this article was to bring together diverse ideas from neuroscience, AI, synthetic biology, evolutionary theory and robotics in order to identify measures and mechanisms that relate to the problem of consciousness. Synergies between these disciplines have started to converge towards a systematic science of consciousness. Following through with these developments, we have attempted to generalize the applicability of current clinical scales of consciousness to artificial agents. In particular, starting from clinical measures of consciousness that calibrate awareness and wakefulness in patients, we have investigated how contemporary AI agents and synthetically engineered organisms compare on homologous measures. An abstraction of processes involving awareness and wakefulness can be generically associated to forms of computational and/or autonomic complexity.

Furthermore, based on insights from evolutionary game theory, we have discussed the function that consciousness serves in nature, and argued that the mechanisms of consciousness arose as an evolutionary game-theoretic strategy. This was why we introduced a third kind of complexity to describe consciousness, namely, social complexity. Social interactions play a crucial role in driving and regulating adaptive responses through behavioral feedback in both natural and artificial systems [20]. In [80,84], it has been suggested that complex social interactions may have evolutionarily served as a trigger for consciousness. For these reasons, social complexity is crucial for constructing a morphospace of consciousness.

A morphospace is a useful construct to study systems-level properties of complex systems based on information-theoretic complexity measures. The three kinds of complexity specified here, capture functional characteristics of biological as well as synthetic complex systems. Using these scales, we have shown how biological organisms including bacteria, bees, C. elegans, primates and humans compare to current AI systems such as deep networks, multi-agent systems, social robots, intelligent assistants such as Siri and computational systems such as IBM’s Watson. Put together, the above three kinds complexity help characterize both, biological and artificial agents in a common framework.

Besides consciousness as we know it (in biology), distinct regions in the morphospace suggest other plausible manifestations of consciousness (based on functional criteria), namely, synthetic, group and simulated consciousness, each based on a distinct embodiment. However, what is far from clear is whether there exist specific thresholds in the values of each complexity, that an agent must surpass in order to attain a level of consciousness. Certainly, from developmental biology we know that both humans (and many higher-order animals) undergo extensive periods of cognitive and social learning, concurrent to physiological development, from infancy to maturation. These phases of physiological, cognitive and social training are necessary for the development of autonomic and cognitive abilities leading to levels of consciousness attained by brains.

Even though we may still be far from understanding most of the engineering principles required to build conscious machines, a complexity-based comparison between biological and artificial systems reveals interesting insights. For example, current AI systems using deep learning tend to cluster along the computational complexity axis of the morphospace, whereas synthetically engineered life forms group closer along the autonomic complexity axis. On the other hand, biologically conscious agents are distributed in regions of the morphospace corresponding to relatively high complexity along all three of the axes (which suggests necessary, if not sufficient, conditions for biological consciousness). In terms of Newell’s criteria, mentioned in the introduction, excluding those criteria that refer exclusively to human-specific traits (language, symbolic reasoning), the remaining ones are completely satisfiable by any agent located in the high complexity region of all three axes of the morphospace. In contrast, current AI or synthetic systems do not check out on this list. Though in 1994 Newell was not explicitly referring to consciousness, it is remarkable to note how those ideas to formulate theories of cognition and intelligence seem to reconcile with current ideas of consciousness. One could summarize the crux of Newell’s criteria as referring to agents displaying autonomous adaptive behaviors with cross-domain problem-solving capabilities, which can be decomposed to the kinds of complexity discussed here.

This perspective on consciousness opens several possibilities for future work. For instance, it may be interesting to further refine the morphospace described here. In particular, computational complexity itself may involve several sub-types involving learning, adaptation, acquiring sensorimotor representations, etc, all of which are relevant for cognitive robotics. Another question arising out of our discussion is whether the emergence of consciousness in a multi-agent social environment can be identified as a Nash equilibrium of a cooperation–competition game. In a game where, say, two species attain consciousness, the population pay-offs in cooperation and competition between them are likely to reach one of possible equilibria due to the recursive nature of social inferences, when an agent attempts to infer the inferences of other agent about its own intentions. Multi-agent models might offer a viable approach to test ideas such as these.

Furthermore, a conceptualization of a morphospace of consciousness offers an interesting comparative perspective on leading candidate theories of consciousness. The main contenders in this case are: (i) Integrated Information Theory (IIT) [10], (ii) Global Workspace Theory (GWT) [8], (iii) Predictive Processing Theories (PPT) [136], (iv) Higher Order Theories (HOT) [137], and (v) Orchestrated Objective Reduction Theory (Orch-OR) [138]. These are also the major theoretical paradigms of consciousness currently being pitted against each other as part of the ‘Structured Adversarial Collaboration Projects’ initiative being supported by the Templeton World Charity Foundation. The ultimate goal (and test) of any theory of consciousness is to satisfactorily explain the so-called “hard problem of consciousness”, that is, ‘How and why first-person phenomenal experiences arise, and what the nature of qualia may be?’ [139]. Our intention, in this work, is not to directly address any of those fundamental questions or propose a new theory of consciousness. Rather, we have investigated a taxonomy of conscious and artificial agents based on complexity, with the objective of highlighting design constraints shared across minds and machines. These constraints may help fine-tune future iterations of the above candidate proposals of consciousness.

Given the morphospace of consciousness, we can now ask the following questoin: What kinds of complexity could the above-mentioned candidate theories of consciousness admit? The first four of these, for the most part, associate consciousness to computational complexity. IIT, with its information-theoretic Φ measure, says little about autonomic or social processes, deferring consciousness to computational mechanisms with high integrated information. GWT explicitly proposes conscious access as a kind of computation [140]. PPT operates within the framework of predictive coding and Bayesian inference. These models are grounded in sensorimotor interactions and, to a certain extent, also involve autonomic processes (see also [141]). In HOT, phenomenal consciousness is postulated to be a higher-order representation of perceptual or quasi-perceptual contents, that is, thoughts or perceptions about first-order mental states. Orch-OR, on the other hand, explicitly states that consciousness is a non-computational process (one that cannot be algorithmically implemented in the Turing sense). This theory associates “proto-consciousness” to an orchestrated objective reduction of the quantum wave-function in dendritic microtubuli. Of the three complexity kinds, processes postulated in Orch-OR belong to the autonomic class and manifest at the molecular level. It is also worth mentioning other non-computational processes such as stochastic dynamics [142] or non-Darwinian mechanisms [143,144] that are relevant to molecular and systems-level biology, but have not yet been fully exploited in the context of consciousness research.

What we have learned here, from synthesizing cross-disciplinary evidence about brains and machines into a unified framework of a morphospace, is that consciousness (at least, as we know it in biology) is supported by processes of at least three kinds of complexity and that these processes are closely intertwined with each other. This poses a challenge for all of the above candidate theories of consciousness. Hence, these theories have to explain how the core mechanisms they associate to consciousness unfold into all three complexity kinds. In the absence of that, the proposed theories at best only describe individuated components of the full problem and may require building bridges with each other in order to reconcile how autonomic, computational and social processes collectively give rise to consciousness.

Related to the above point, the morphospace, described here, suggests a taxonomy of complexity into three kinds of systems-level processes. In a sense, these correspond to the brain ($C_{Computational}$), body ($C_{Autonomic}$) and environment ($C_{Social}$). While this taxonomy was derived from functional arguments (the H5W problem), this correspondence to the brain, body and environment suggests architectural constraints on conscious agents, namely, that such architectures include computational, embodied, situated and social modalities. However, this by itself is not surprising; there is extensive work in the cognitive science literature studying each of these paradigms, some of which also concern the easy problem versus hard problem dichotomy [139]. The point we emphasize in this work is that a morphospace forces one to think of these functions and design constraints in a common framework. It is also a challenge for all existing theories of consciousness to show how the axioms they propose to address the “hard” problem reconcile with the integration of the “easy” problems via interactions between the brain, body and environment. This is the issue that a morphospace of consciousness brings to the forefront.

As a final remark, note that the taxonomy of the three complexity kinds discussed here, also shows up in current AI architectures where physics and psychology engines provide priors to hierarchical Bayesian networks used for meta-learning or learning to learn [145]. The physics engine in this case can be identified to processes mostly along the autonomic axis. The psychology engine largely accounts for social processes. Computational and reasoning processes are implemented on Bayesian inference engines. Of course, these engines do not operate mutually exclusively, being closely coupled and co-ordinated with each other. This observation lends evidence to the claim that a morphospace, as we have described here, serves as a useful construct for identifying general design principles and constraints in our theories and architectures of biological as well as artificial intelligence.

## Figures and Tables

**Table 1:** Presented in the table below is a classification of three kinds of complexity relevant for charting a taxonomy of consciousness, namely, autonomic, computational and social complexity. This classification is based on the respective building blocks or substrates of each complexity kind, the systems-level realizations of these substrates, and their associated emergent phenomena.

	$C_{\mathrm{Autonomic}}$	$C_{\mathrm{Computational}}$	$C_{\mathrm{Social}}$
Building Blocks	Sensors, Actuators	Neurons, Transistors	Individual Agents
Systems-Level	Prokaryotes, Autonomic	Cognitive Systems,	Population of Agents,
Realizations	Nervous System, Bots	Brains, Microprocessors	Social Organizations
Emergent	Self-Regulated	Problem Solving	Signaling Conventions,
Phenomena	Real-Time Behavior	Capabilities	Language, Social Norms,
			Arts, Science, Culture

**Table 2:** A summary of complexity measures that have been tested on various autonomic, computational and social systems.

Complexity Kind	Complexity Measures
$C_{Autonomic}$	Index of Autonomous Functioning
	Synergistic Information
	Morphological Computation
$C_{Computational}$	Integrated Information (v1, v2, v3, geometric)
	Stochastic Information/Total Information Flow
	Mutual & Specific Information
	Redundant & Unique Information
	Synergistic Information
	Transfer Entropy & Information Transfer
$C_{Social}$	Collective Intelligence Factor
	Integrated Information v2

## Data Availability

No new data created or used in this study.
